# Facile fabrication of Eu-based metal–organic frameworks for highly efficient capture of tetracycline hydrochloride from aqueous solutions

**DOI:** 10.1038/s41598-023-38425-x

**Published:** 2023-07-10

**Authors:** Xue He, Yong Liu, Qicui Wang, Tao Wang, Jieli He, Anzhong Peng, Kezhen Qi

**Affiliations:** College of Pharmacy, Dali University, Dali, 671003 People’s Republic of China

**Keywords:** Chemistry, Materials science, Nanoscience and technology

## Abstract

The tetracycline hydrochloride (TCH) removal from wastewater is important for the environment and human health yet challenging. Herein, the Eu-based MOF, Eu(BTC) (BTC represents 1,3,5-trimesic acid) was prepared by an efficient and environmental-friendly strategy, and then was used for the TCH capture for the first time. The Eu(BTC) was characterized by different methods such as X-ray diffraction, scanning electron microscopy and Fourier-transform infrared spectroscopy. The TCH uptake of Eu(BTC) was investigated systematically. The influences of experiment conditions such as solution pH value, adsorption time and initial concentration on TCH capacity of Eu(BTC) were also studied. The Eu(BTC) obtained exhibited remarkable TCH uptake (q_m_ was up to 397.65 mg/g), which was much higher than those of most materials such as UiO-66/PDA/BC (184.30 mg/g), PDA-NFsM (161.30 mg/g) and many carbon-based materials reported till now. Besides, the TCH adsorption behavior on Eu(BTC) was explored by Freundlich and Langmuir equations, and the adsorption mechanism was further analyzed. The experimental results suggested that the TCH adsorption mechanism of Eu(BTC) included the π–π interaction, electrostatic interaction and coordinate bonds. The excellent TCH adsorption performance and the efficient fabrication strategy make the Eu(BTC) prepared promising in TCH removal.

## Introduction

Nowadays, the aquatic environment pollutions caused by heavy metal ions^1^, organic pollutants^2^ and antibiotics^3^ have become increasing global issues. Particularly, as the most commonly used and highly effective pharmaceutical component, antibiotics are extensively involved in the agricultural industry and human therapy^4^. It’s worth noting that a large proportion of antibiotics are not absorbed completely by human body and animals, and then excreted into the ecosystem as metabolites or even the primitive state^5^. The antibiotics discharged are mainly from the agricultural, hospitals, aquaculture farm, and industrials effluents^6–9^. It has been reported that the concentration of antibiotics can reach up to 100–500 mg/L in pharmaceutical and medical wastewater^10,11^. Excessive antibiotics emission would inevitably create the severe threat to human survival and environmental safety. The most typical and representative antibiotic, tetracycline hydrochloride (TCH), exhibits middle water solubility (231 mg/L)^12^, durability and high biotoxicity, and is usually detected in water environment.

Due to the deep removal of TCH from aqueous solution is difficult for the traditional sewage treatment technology, and the TCH are prone to accumulate in soil, groundwater and surface water. Development of an efficient strategy to remove the antibiotics remains a pronounced challenge^13–16^. Recently, different methods have been reported for the TCH removal such as electrolysis^17^, oxidation^18^, photochemical degradation^19^ and adsorption^20^. Among these methods, the adsorption technology is highly regarded as the first choice for antibiotics capture because of its advantages such as high energy efficiency, simple operation and environmental-friendliness^21–23^. While most adsorbents reported displayed inferior adsorption selectivity and capacity till now, and it is urgent to develop high-performance adsorbents^24^. To the best of our knowledge, some porous materials have been exploited to remove TCH from waste water including metal organic frameworks (MOFs), lignocellulosic materials^25^, kaolin^26^, porous carbon^27^ and metal oxides^28^.

Among the adsorbents mentioned above, MOFs are composed of divergent metal ions or clusters and organic ligands^29–32^, and demonstrate unique properties such as high surface area, tunable pore size, and tailorable functionalities, which are unmatched by conventional materials. However, most MOFs often have low stability in aqueous solution, and their applications are usually focused on the adsorption of organic molecules, drug delivery vectors, luminescence and catalysis^33–35^. There have been few reports about the antibiotic removal using lanthanide MOFs to date^36^. In this work, an eco-friendly MOF, Eu(BTC) (BTC means 1,3,5-trimesic acid), was prepared by a facile strategy, and employed for the TCH removal from aqueous solution for the first time. The TCH adsorption kinetics and adsorption isotherms of Eu(BTC) were examined in details. Besides, the influence of pH value in solution, adsorption time and beginning concentration on the TCH adsorption performance of Eu(BTC) were also fully studied. The adsorption data were fitted with Freundlich and Langmuir equations to investigate the behavior of TCH on Eu(BTC). The experimental results indicated that the TCH adsorption capacity of Eu(BTC) was mainly depended on the synergetic effect of the π-π interaction and chemisorption. The reusability and stability of Eu(BTC) in water were studied according to the literatures reported^37–40^. The Eu(BTC) prepared may offer a promising alternative for the antibiotic removal from waste water.

## Experimental

### Chemicals

*N*,*N*-Dimethylformamide (C_3_H_7_NO, 99.8%), 1,3,5-trimesic acid (C_9_H_6_O_6_, 99%), europium nitrate hexahydrate (Eu(NO_3_)_3_·6H_2_O, 98%), tetracycline hydrochloride (C_22_H_25_ClN_2_O_8_, 97%), sodium acetate anhydrous (CH_3_COONa, 99.0%). All reagents purchased from J & K Scientific Ltd. or Acros Organics were used without further purification and all solutions were obtained by successive dilutions of the stock solution. There are no direct human/human samples are involved in this study.

### Synthesis of Eu(BTC)

Eu(BTC) was synthesized by solvothermal reaction. Briefly, 0.033 g Eu(NO)_3_·6H_2_O and 0.016 g H_3_BTC were placed into 10 mL DMF to acquire the reaction precursor, 0.02 g CH_3_COONa was dissolved in the blended solution (DMF:C_2_H_6_O:H_2_O = 3:2:2, v/v) in advance, and then the reaction precursor and blended solution were put into a round-bottomed flask, heated and accompanied with continuous stirring at 80 °C for 24 h. When the reaction finished, the flask was cooled to room temperature. The resulting residue was washed continuously with anhydrous ethanol and deionized water until the colorless product was observed, and then the sample was dried at 60 °C overnight to obtain the target Eu(BTC).

### Characterization of Eu(BTC)

The specific surface area, pore diameter determination and pore volume were calculated by the BET methods (Quantochrome NOVA, USA). The powder X-ray diffraction pattern of the Eu(BTC) was acquired by powder X-ray diffractometer (Bruker D8, Germany). The FT-IR spectra of Eu(BTC) was collected based on a FT-IR spectrometer in the 400–4000 cm^−1^ range (NICOLET 380, USA). elemental composition of the samples was characterized by X-ray photoelectron spectroscopy (ESCALAB 250Xi spectrometer, USA). Scanning electron microscopy was applied to observe the morphologies and structures of the products (FEI quanta 400feg, USA). The optical property of samples was investigated using an ultraviolet–visible spectrophotometer (UV–Vis, TU-1901, USA).

### Adsorption experiments

The adsorption capacity of Eu(BTC) was performed by batch sorption experiments. The influence of experimental conditions such as solution pH (2–10), adsorption time (2–30 h), temperature (298–328 K) and TCH initial concentration (40–140 mg/g) on the adsorption performance was evaluated, respectively. In a typical adsorption procedure, the TCH was dissolved in ultrapure water to obtain the stock solutions (200 mg/L). Standard solutions (20–140 mg/L TCH) were prepared by diluting the stock solutions. All the adsorption time was 24 h, ensuring the establishment of the adsorption equilibrium. The residual amount of TCH in the solution was analyzed by UV–Vis absorption spectroscopy at 357 nm.

#### Adsorption isotherms

5 mg Eu(BTC) was placed into 10 mL solution with different TCH concentrations (20, 40, 60, 80, 100, 120 and 140 mg/L), respectively. After 24 h, at 298, 303, 313, 323 and 333 K, 5 mL solution of each sample was periodically taken out for analysing the TCH concentration remained. The calibration curve was acquired according to the spectrum of the standard TCH solutions. The TCH adsorption capacity of Eu(BTC) was calculated using the Eq. (1).

1 $$q_{e} = \frac{{(C_{0} - C_{e} ) \cdot V}}{m}$$

where q_e_ (mg/g) means the TCH amount adsorbed when reaching the adsorption equilibrium, C_0_ (mg/L) is the TCH initial concentration and C_e_ (mg/L) is the equilibrium concentration, m (g) means the dosage of Eu(BTC) and V (L) represents the TCH solution volume used.

#### Adsorption kinetics

The adsorption kinetics was investigated at the predetermined time interval from 2 to 30 h. 50 mg Eu(BTC) was dispersed into 100 mL TCH solution (100 mg/L) at 298 K. 5 mL solution was sampled at definite time interval and then was analyzed by spectrophotometer.

#### Influences of pH

5 mg Eu(BTC) was immersed into different TCH solutions (10 mL, 100 mg/L) with pH from 2 to 10. The pH in aqueous solution was adjusted with dilute HCl or NaOH solution. After reaching the adsorption equilibrium, the residual TCH concentration was determined by UV spectrophotometer, respectively.

#### Influences of ionic strength

The influences of ionic strength on TCH adsorption was studied. 20 mg Eu(BTC) was placed in the 50 mL, 50 mg/L TCH solution containing 0.01–0.08 mol/L NaCl.

## Results and discussion

### Characterization

#### BET analysis

**Figure 1:**
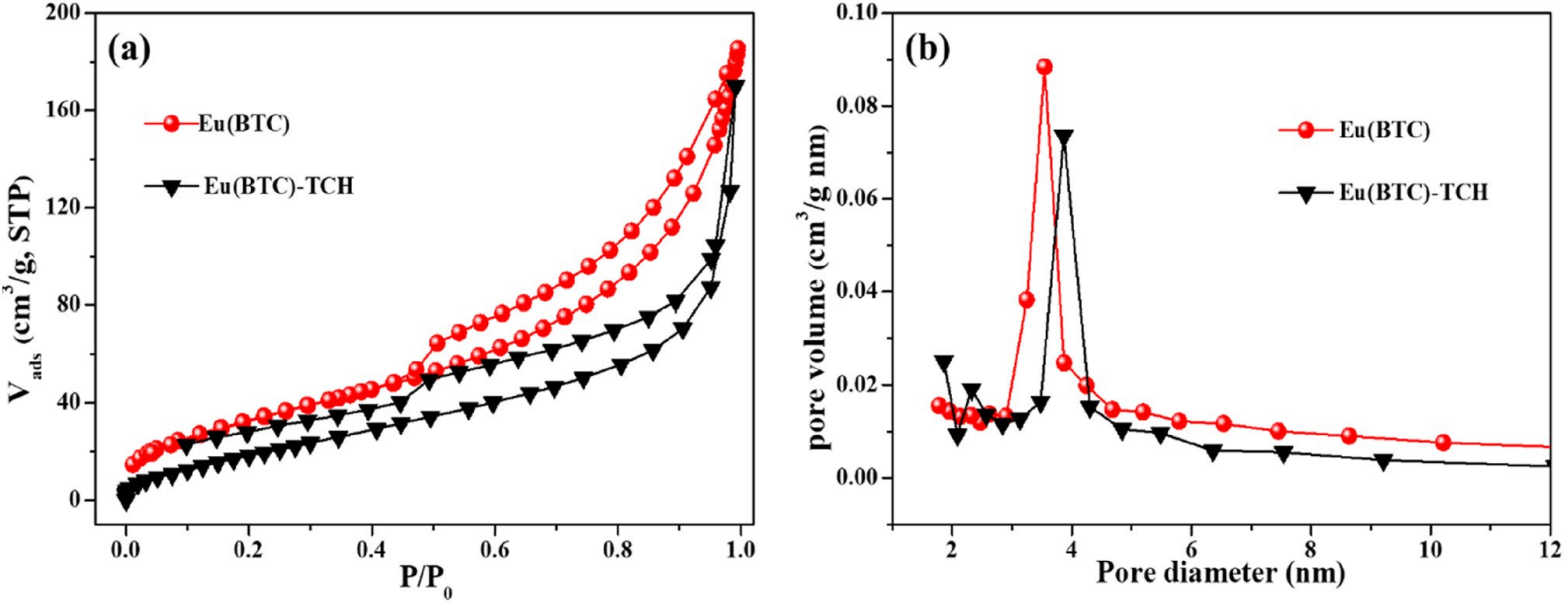
(**a**) Nitrogen adsorption–desorption isotherm and (**b**) pore size distribution of Eu(BTC) before and after TCH adsorption.

For examining the porosity of Eu(BTC), the nitrogen adsorption/desorption isotherms were measured. The nitrogen adsorption–desorption curve and pore size distribution curve of Eu(BTC) before and after TCH adsorption were shown in Fig. 1. It can be seen that the Eu(BTC) displayed a good nitrogen adsorption ability at the relative pressure (*P/P*_*0*_) from 0 to 1, and the nitrogen isotherms was consistent with the representative type IV isotherm, which suggested the existence of mesopores in Eu(BTC). As shown in Table 1, the calculated surface area (*S*_*BET*_) of Eu(BTC) was 123.87 m^2^/g and the average pore diameter was 8.81 nm. Compared with the Eu(BTC), the *S*_*BET*_ and pore volume of Eu(BTC)-TCH (after TCH adsorption) significantly decreased, which were attributed to the pore filling effect in the process of TCH adsorption^41^.

**Table 1:** *S*_*BET*_, pore size and volume of Eu(BTC).

Adsorbents	Porous volume (cm^3^/g)	*S*_*BET*_ (m^2^/g)	Average pore diameter (nm)
Eu(BTC)	0.273	123.865	8.806
Eu(BTC)-TCH	0.258	80.605	7.789

#### XRD analysis

**Figure 2:**
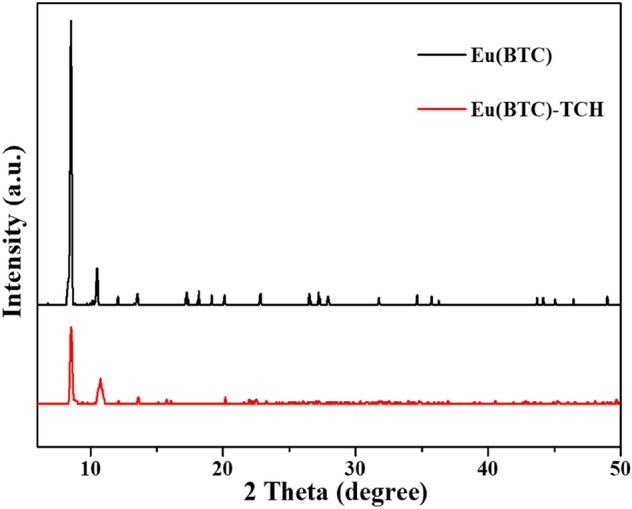
XRD pattern of Eu(BTC) before and after TCH adsorption.

The XRD measurement was carried out to investigate the crystalline structure of Eu(BTC) before and after TCH adsorption. The characteristic diffraction peaks of Eu(BTC) were displayed in Fig. 2. The diffraction pattern exhibited two distinct peaks at 8.54° and 10.50°, which was agreed with the simulated patterns reported^42^. The diffraction peaks were narrow and strong, indicating the excellent crystallinity of Eu(BTC) obtained. In addition, there was almost no impurity peaks appeared in the diffraction pattern, suggesting the ideal purity and crystallinity of the Eu(BTC). The position of diffraction peaks remained unchanged before and after TCH adsorption, conforming the perfect stability of Eu(BTC).

#### FT-IR analysis

**Figure 3:**
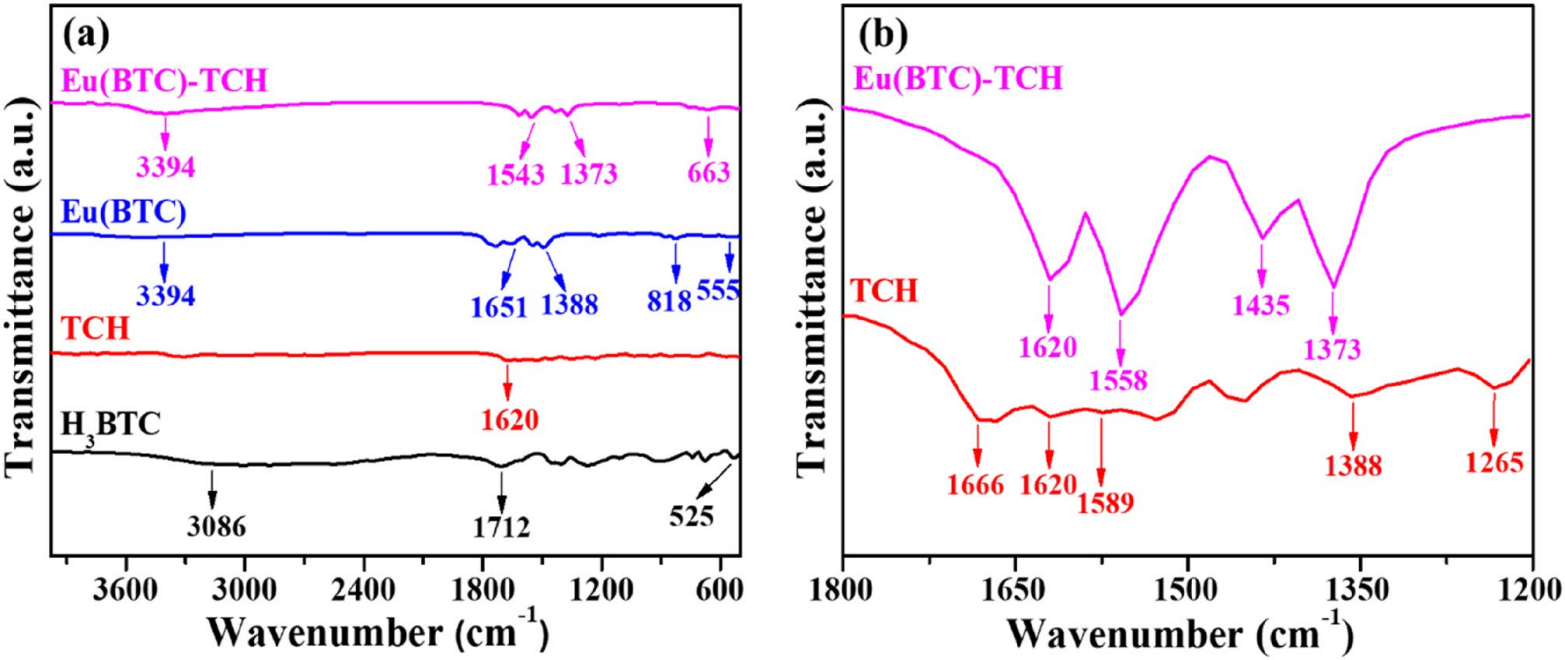
(**a**) FT-IR spectra of H_3_BTC, TCH, Eu(BTC), Eu(BTC)-TCH, and (**b**) the FT-IR spectra of Eu(BTC)-TCH and TCH from 1200 to 1800 cm^−1^.

The FT-IR spectra of Eu(BTC) before and after the TCH adsorption, H_3_BTC and TCH were measured. As shown in Fig. 3, H_3_BTC displayed three characteristic peaks at 3086 cm^−1^ (the stretching vibration peak of –OH), 1712 cm^−1^ (the stretching vibration peak of –C=O) and 525 cm^−1^ (the bending vibration peak of –C=O). The above three characteristic peaks disappeared in the spectrum of Eu(BTC), indicating that the BTC ligands were completely deprotonated after the reaction. The peaks of Eu(BTC) at 1543–1651 and 1373–1388 cm^−1^ can be allocated to the stretching vibrations and the bending vibrations of C=O, respectively. The broad band at 3394 cm^−1^ was assigned to the hydrogen-bonded vOH groups from the water adsorbed, suggesting that the water molecules were successfully coordinated with Eu^3+^. These results demonstrated the successful synthesis of Eu(BTC). In the case of TCH, the peaks at 1589, 1620 and 1666 cm^−1^ could be assigned to the stretching vibrations of –C=O in ring C, ring A and amide I (the C=O group of the –CONH_2_), respectively. Compared with that of Eu(BTC), the main absorption peaks of Eu(BTC)-TCH kept almost unchanged, while the wavenumber migration occurred to some extent. For the Eu(BTC)-TCH, the peak at 1620 cm^−1^ could be attributed to TCH, meaning the successful TCH adsorption of Eu(BTC). The peaks of Eu(BTC) at 3394, 1651 and 818 cm^−1^ were all significantly weakened, confirming the TCH absorption of Eu(BTC) again. The strong peak at 818 cm^−1^ was ascribed to C–H bending vibrations of benzene, indicating the existence of aromatic carbon in Eu(BTC), and giving evidence of the π–π* interactions about the TCH adsorption of Eu(BTC).

#### XPS analysis

**Figure 4:**
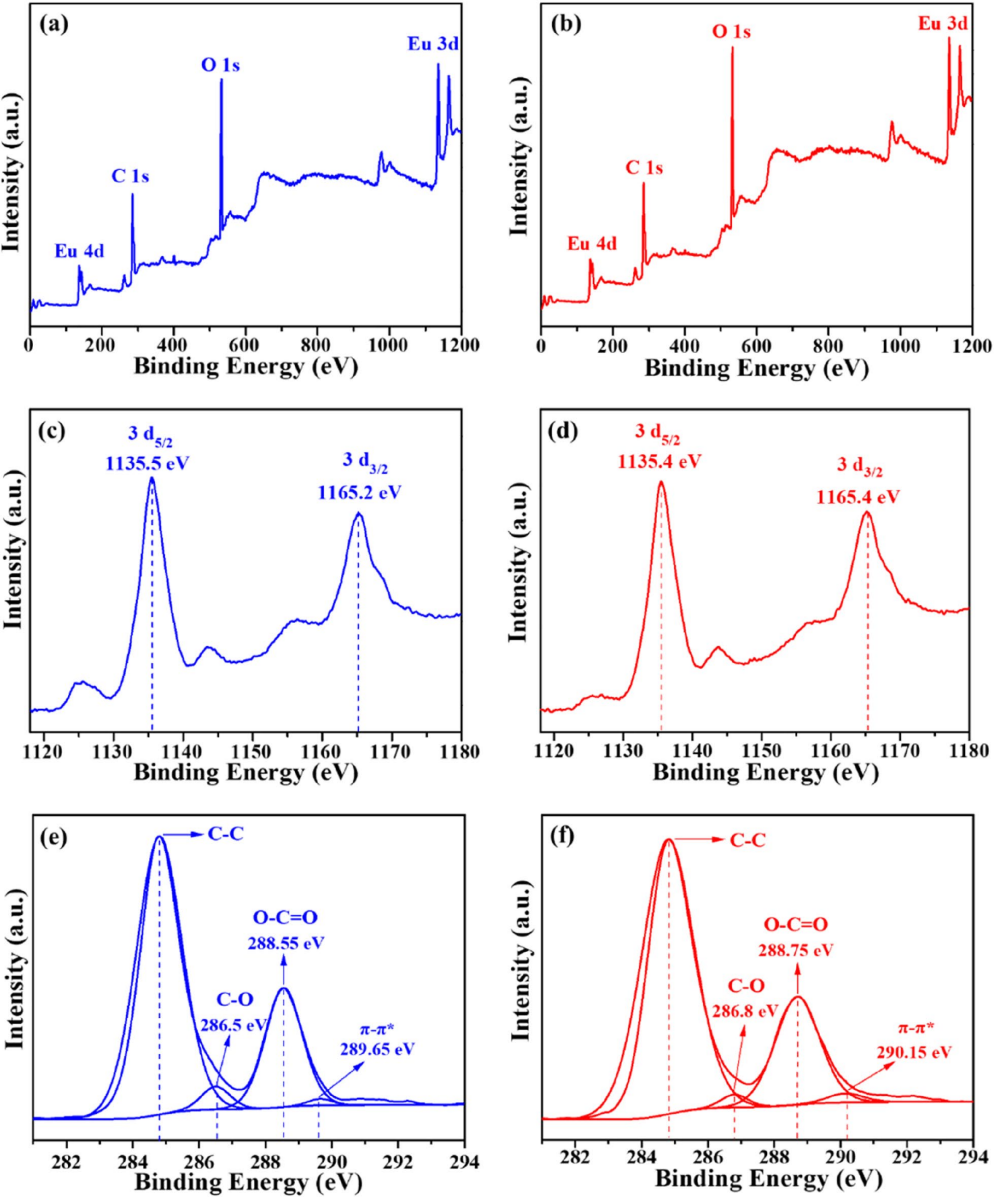
XPS spectra of Eu(BTC) (**a**, **c**, **e**) and Eu(BTC)-TCH (**b**, **d**, **f**).

The X-ray photoelectron spectroscopy (XPS) analysis was performed to investigate the composition and chemical status of Eu(BTC). The full spectrum of Eu(BTC) and Eu(BTC)-TCH displayed that the primary chemical components were C, O and Eu (Fig. 4a,b). As shown in the detailed spectra of Eu 3*d* (Fig. 4c,d), the peak at 1165.2 eV could be corresponded to the Eu 3*d*_3/2_, the peak at 1135.5 eV was assigned to the Eu 3*d*_5/2_ of Eu(BTC), and the peak at 1135.4 eV was ascribed to the Eu 3*d*_5/2_ of Eu(BTC)-TCH^43^. The C 1*s* spectrum (Fig. 4e,f) of Eu(BTC) before and after TCH adsorption exhibited four peaks centered at 289.65–290.15 (π–π*), 288.55–288.75 (C=O), 286.50–286.8 (C–O) and 283.4–238.5 eV (C–C). All the C 1*s* peaks shifted to higher binding energy after the TCH adsorption, indicating that these functional groups might improve the TCH adsorption.

#### SEM analysis

**Figure 5:**
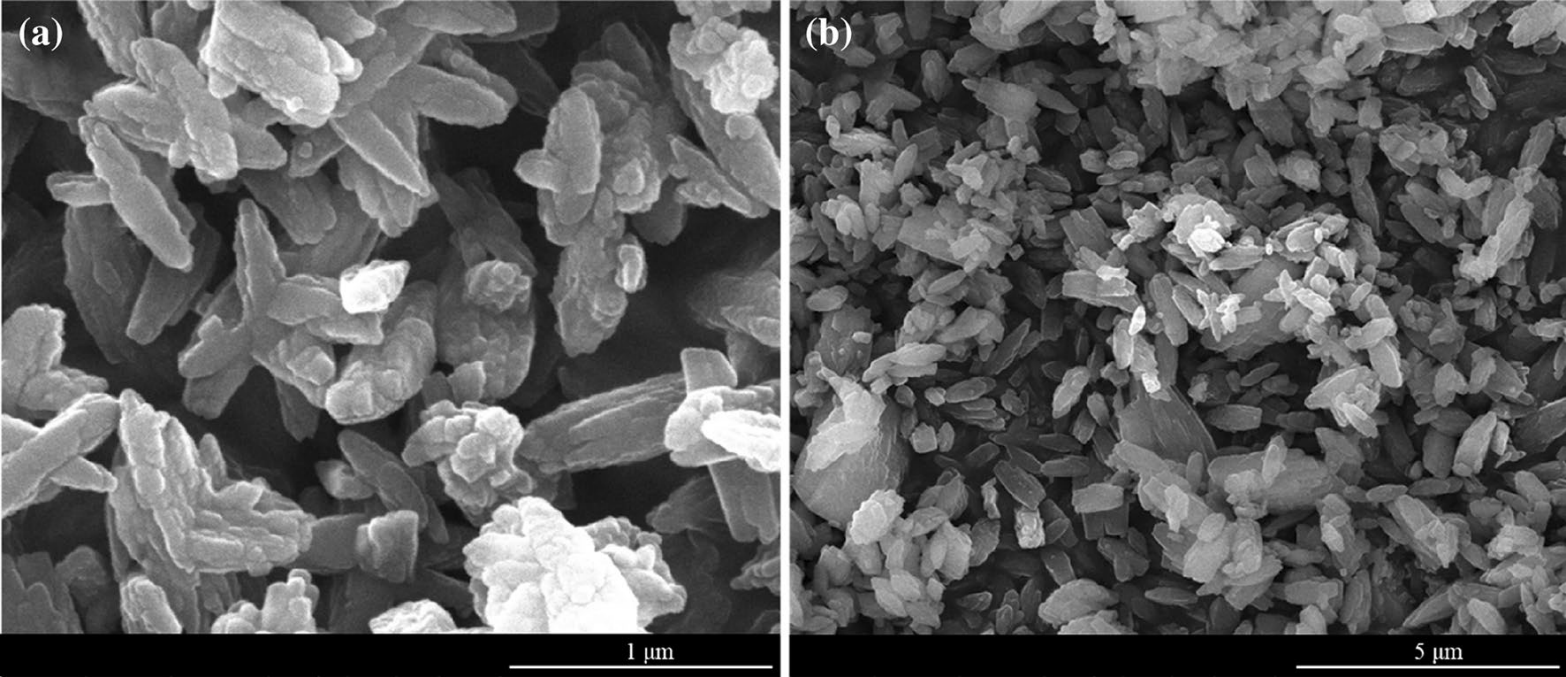
SEM images of Eu(BTC).

The structure and morphology of Eu(BTC) were observed by scanning electron microscopy (SEM). As displayed in Fig. 5, the overall appearance of Eu(BTC) was rod-shaped, and the particles were reunited and gathered into clusters. Part of the boundaries between particles disappeared and fused into larger particles, and irregular deformation appeared for some particles.

### Batch experiments

#### Effect of pH

**Figure 6:**
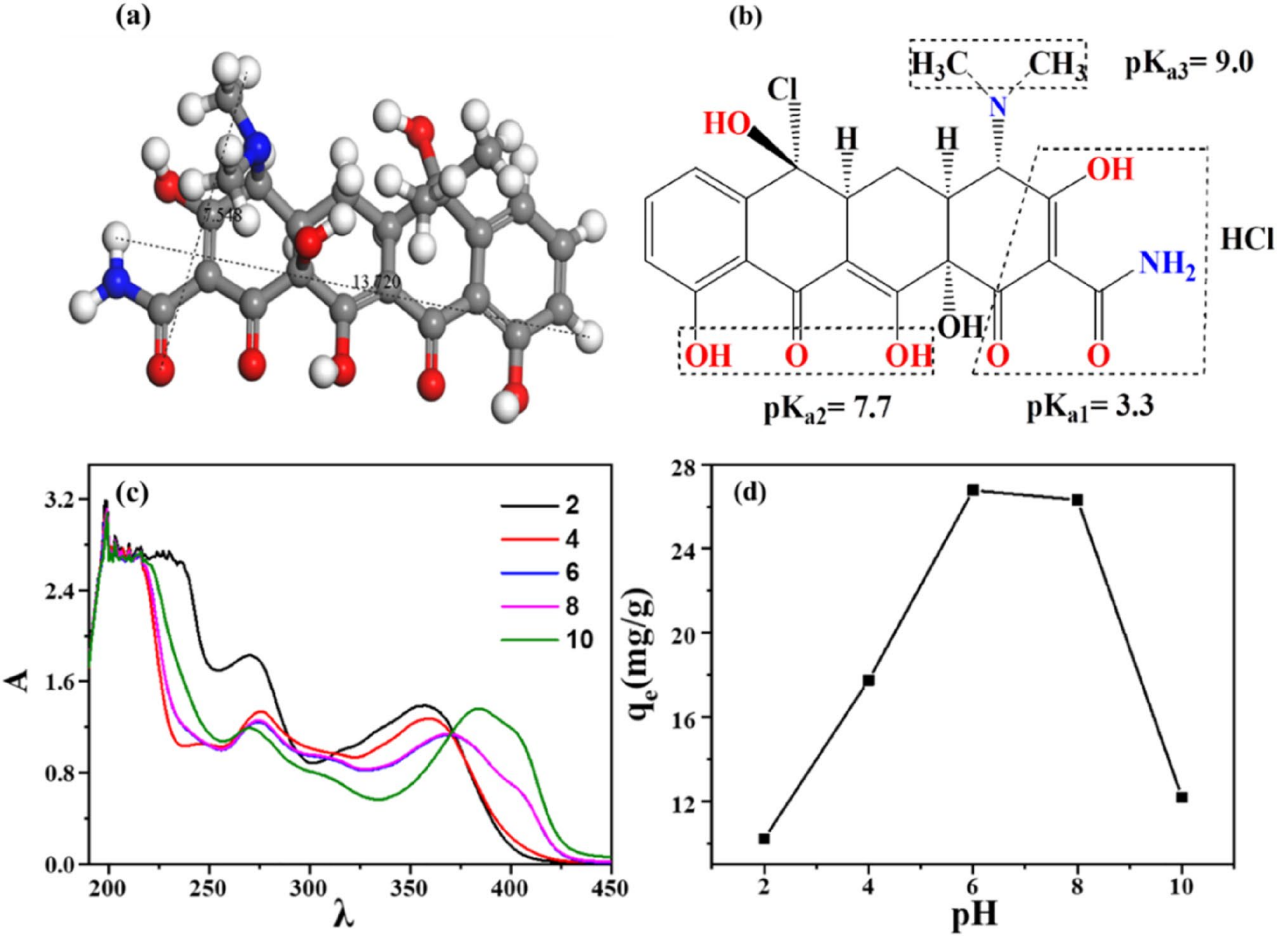
(**a**) Molecular size of TCH (Color code: C, gray; O, red; H, white; N, blue), (**b**) Molecule structure of TCH, (**c**) UV–Vis spectra of the TCH adsorption on Eu(BTC), and (**d**) pH effect on TCH adsorption of Eu(BTC).

**Figure 7:**
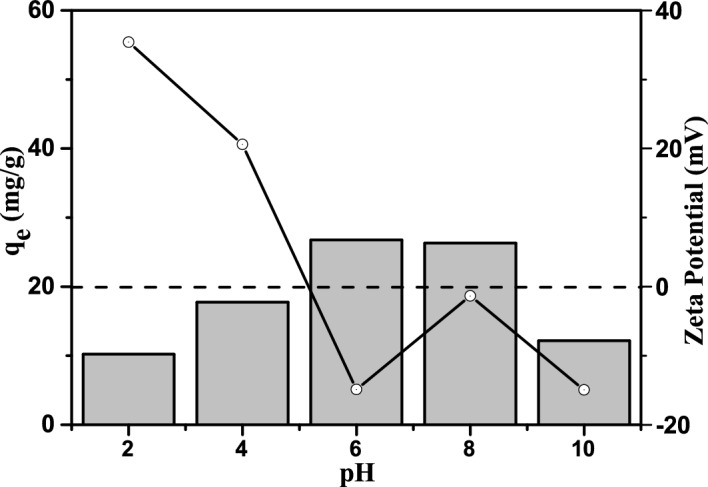
Effect of solution pH and zeta potential on TCH adsorption of Eu(BTC). (C_0_ = 100 mg/L, T = 298 K).

The pH value affects seriously the ionization of TCH in aqueous solution, and then leading to non-negligible influence on the TCH adsorption. The effect of pH (2 ~ 10) on TCH uptake was studied. The TCH is amphoteric molecule and can form different ionizing functional groups in aqueous solution through protonation-deprotonation reaction (Table 2)^44^. The anionic content of TCH will increases with the rise of pH value, which benefits the TCH adsorption because of the strong electrostatic attractions^45^ and the cations of the adsorbent in solution will reduces with the increasing pH^46^. As shown in Fig. 6c and d, the TCH adsorption amount of Eu(BTC) increased quickly when the pH value changed from 2 to 6, and then leveled off at pH 6 ~ 8. The TCH uptake decreased rapidly when the pH value rose further. For investigating the TCH adsorption mechanism, the zeta potential changes of Eu(BTC) were measured and illustrated in Fig. 7. When the pH value was lower than 3.3, the repulsive interaction caused by the positively charged TCH^+^ and the positive groups of Eu(BTC), afforded a poor TCH adsorption. At about pH 3.3 ~ 7.7, the TCH remained the zwitterions form, and its adsorption amount did not decline with the pH increase, indicating that the repulsive interaction was not the main factor in the adsorption process. At pH 6, the Eu(BTC) demonstrated the largest adsorption capacity, which can be attributed to the π–π interactions of benzene rings and coordinate bonds. In addition, When the pH value was higher than 7.7, the negative charge of TCH (TCH^−^ and TCH^2–^) and OH^−^ increased gradually, the Eu(BTC) contained abundant anions, thus, the strong repulsive force between the TCH and the Eu(BTC) caused a low TCH adsorption. While the adsorption capacity decreased, plenty of TCH adsorbed remained, possibly attributing to the pore filling.

**Table 2:** Ionizable functional groups of TCH in different pH.

	pH value	Existence form	Existence form
TCH	pH < 3.3	H_4_TC^+^	Cations
	3.3 < pH < 7	H_3_TC	Neutral form
	7.7–9.0	HTC^−^	Anions
	pH > 9.0	HTC^2−^

#### Effect of ionic strength

The effect of ionic strength on TCH adsorption of Eu(BTC) was examined. As displayed in Fig. S1, Na^+^ did not significantly affect the TCH adsorption of Eu(BTC) (M ≤ 0.02). However, with the increasing of Na^+^ concentration from 0.06 to 0.08 mol/L, the TCH adsorption amount decreased, which could be ascribed to the competitive adsorption between the Na^+^ and TCH on Eu(BTC) via electrostatic interaction.

#### Effect of adsorption time: adsorption kinetics

**Figure 8:**
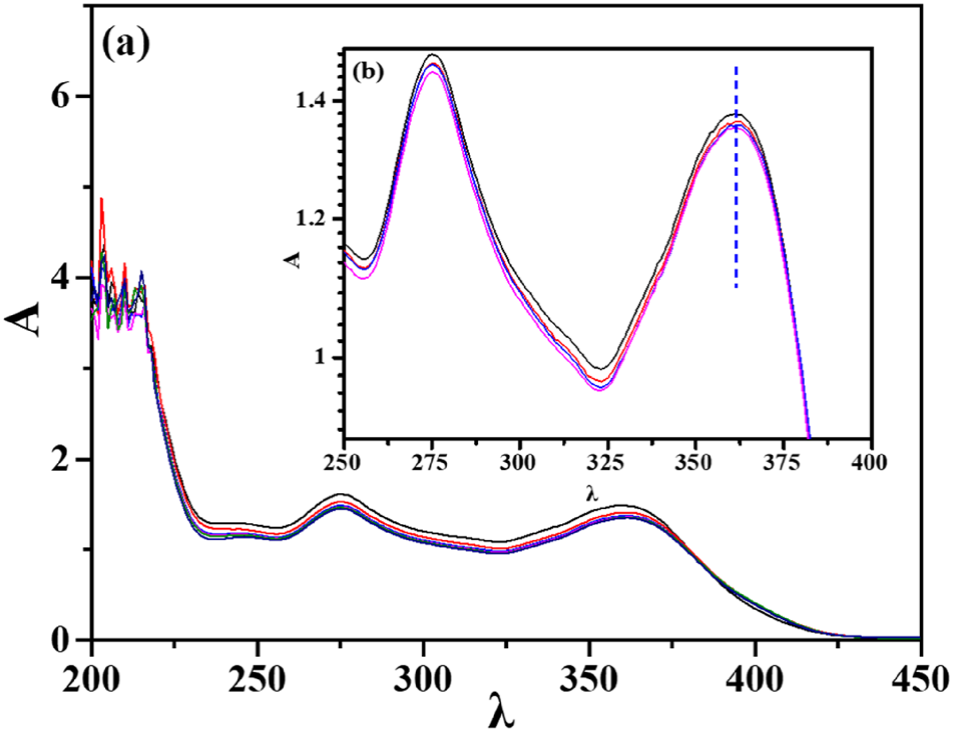
(**a**) UV–Vis spectra of TCH adsorption at different time (C_0_ = 50 mg/L, T = 298 K) and (**b**) UV–Vis spectra from 250 to 400 nm (Black: 4 h, A = 1.615; Red: 8 h, A = 1.53; Blue: 12 h, A = 1.489; Rose red: 16 h, A = 1.471; Green: 20 h, A = 1.467; Dark blue: 24 h, A = 1.455).

**Figure 9:**
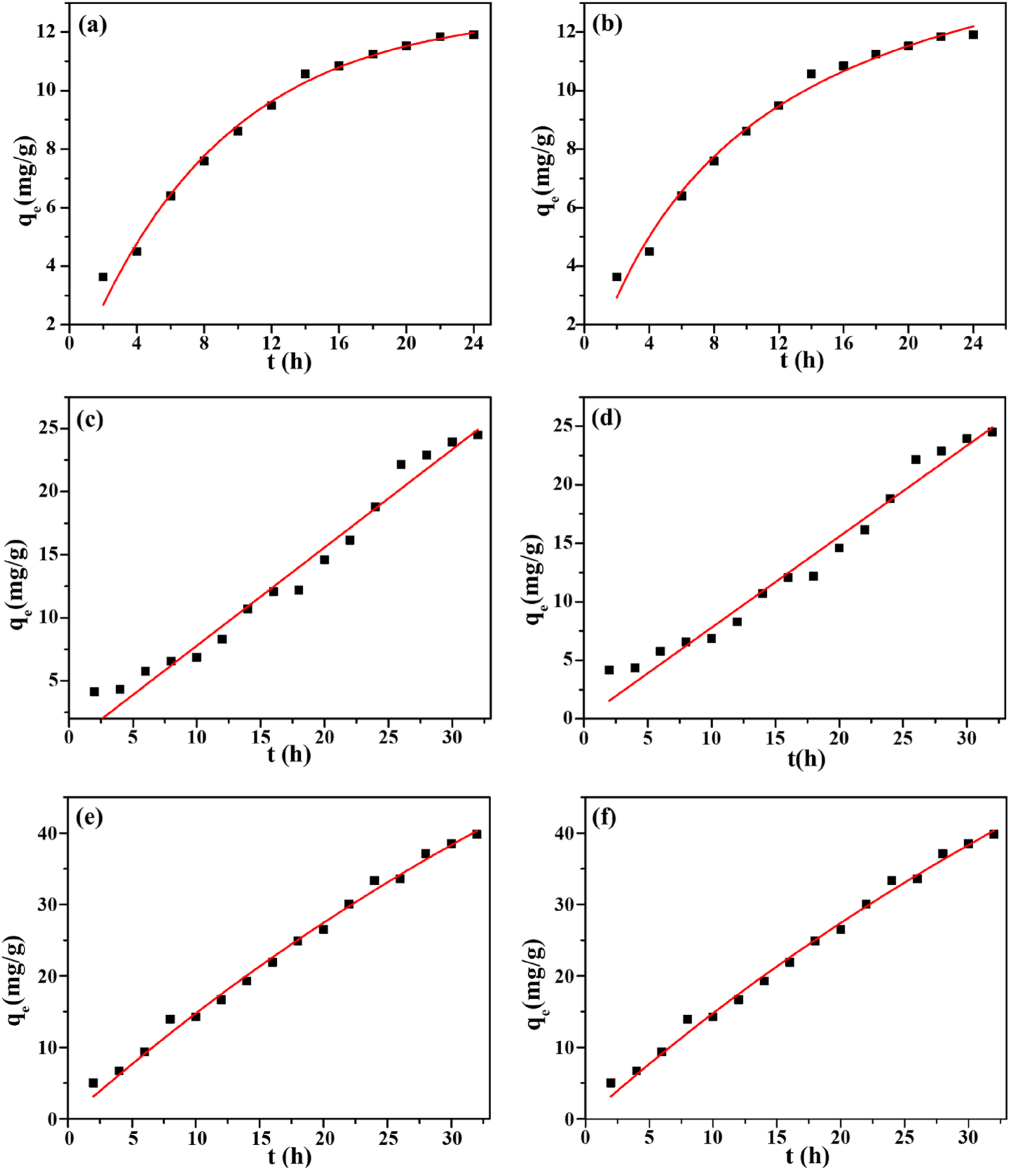
The pseudo-first order plots (**a**, 298 K; **c**, 323 K; **e**, 333 K); the pseudo-second order plots (**b**, 298 K; **d**, 323 K; **f**, 333 K) (C_0_ = 100 mg/L, m = 5 mg, V = 10 mL).

The fast adsorption rate and high adsorption capacity are critical for an ideal adsorbent. The TCH adsorption kinetics of Eu(BTC) were examined, the adsorption experiments at different time (2, 4, 6, 8, 10, 12, 14, 16, 18, 20, 22, 24, 26, 28 and 30 h) were conducted. The UV–vis spectra of TCH adsorption at different time was presented in Fig. 8. To explore the adsorption process, all the experimental results were matched with the pseudo-first-order (Fig. 9a,c,e) and the pseudo-second-order kinetic (Fig. 9b,d,f) and the fitting parameters obtained were enumerated in Table 3. The fitting parameters was calculated according to the following equation:

**Table 3:** Nonlinear fitting kinetic parameters of TCH adsorption on Eu(BTC).

T (K)	Pseudo-first-order kinetic model			Pseudo-second-order kinetic model		
	k_1_ (h^−1^)	q_e_ (mg/g)	R^2^	K_2_	q_e_ (mg/g)	R^2^ (mg/g)
298 K	0.1177	12.7	0.9857	0.0061	17.10	0.9864
333 K	0.0153	104.2	0.9925	3.9E^-5^	186.78	0.9926

The pseudo-first-order equation:

2 $$\ln \left( {q_{e} - q_{t} } \right) = \ln q_{{e\left( {\exp } \right)}} - K_{1} t$$

The pseudo-second-order equation:

3 $$\frac{t}{{q_{t} }} = \frac{1}{{k_{2} q_{{e\left( {\exp } \right)}}^{2} }} + \frac{t}{{q_{{e\left( {\exp } \right)}} }}$$

where q_t_ (mg/g) represents the TCH amount adsorbed of Eu(BTC) at time t, k_1_ (h^−1^) is the constant of pseudo-first-order model and k_2_ (g/mg min) means the equilibrium pseudo-second-order rate constant.

In the initial stage, the adsorption rate increased with the increasing adsorption time, and then slowed down as the time proceeded. The fast TCH adsorption rate in the beginning was ascribed to the abundant active sites in the unoccupied surface of Eu(BTC). The laboratory data were fitted with both the pseudo-first-order model and pseudo-second-order model (0.90≦R^2^≦0.99). The R^2^ values of the pseudo-second-order model were in the range of 0.9864 ~ 0.9926, which were higher than that of the pseudo-first-order model (0.9857 ~ 0.9925), confirming the existence of chemisorption in the TCH adsorption of Eu(BTC). Whereas, as for the adsorption rate constant k_2_, an opposite result was obtained, suggesting that the fast adsorption plays a leading role in TCH adsorption by Eu(BTC)^47^.

### Effect of initial TCH concentration: adsorption isotherms

The adsorption isotherm was of great importance in assessing the maximum adsorption capacity and providing insight into the adsorption mechanism. To investigate the interaction between adsorbate and adsorbent, the adsorption equilibrium data of Eu(BTC) at different initial concentrations (20 ~ 140 mg/L) were simulated with two usually used isotherm models, Langmuir and Freundlich models. The TCH adsorption isotherms of Eu(BTC) were studied at 298, 303, 313, 323 and 333 K. The Langmuir, Freundlich and Dubinin–Radushkevich isotherm models were expressed as Eqs. (4), (5) and (6), respectively.

Langmuir:

4 $$\frac{{C_{e} }}{{q_{e} }} = \frac{1}{{q_{m} k_{L} }} + \frac{{C_{e} }}{{q_{m} }}$$

Freundlich:

5 $$\ln q_{e} = \ln k_{F} + \frac{1}{n}\ln C_{e}$$

Dubinin–Radushkevich:

6 $$_{{q_{e} = q_{m} e^{{ - \beta \varepsilon^{2} }} }}$$

where q_m_ (mg/g) represents the theoretical maximum adsorption amount, k_L_ refers to the constant of Langmuir equilibrium adsorption, k_F_ is empirical constant that represents the Freundlich constants, n is the no-linearity constant^48,49^, respectively. The parameters of these adsorption isotherms were listed in Table 4 in details. β (mol^2^/KJ^2^) is the D–R constant, ɛ (KJ^2^/mol^2^) is the polanyil potential, and q_m_ is the adsorption capacity.

**Table 4:** Linear fitting parameters for TCH adsorption of Eu(BTC) by the Langmuir and Freundlich models.

T (K)	Langmuir model			Freundlich model		
	K_L_	q_m_ (mg/g)	R_L_^2^	1/n	K_F_	R_F_^2^
303 K	0.00467	397.65 (5.35)	0.9381	0.3119	1.1089	0.9404
313 K	0.0141	191.73 (0.14)	0.78105	0.8846	3.7258	0.7878
323 K	0.0183	120.29 (9.28)	0.9498	0.6520	4.36803	0.9817

Values in parentheses represent standard deviations.

**Figure 10:**
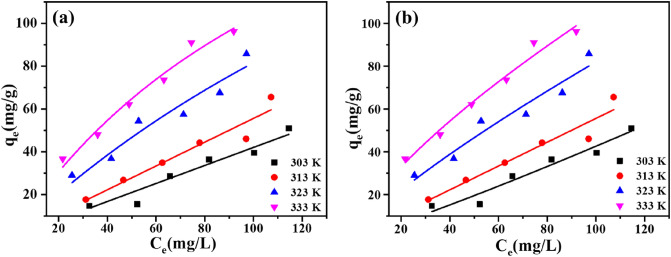
(**a**) Isotherm model fitting curves of Langmuir model and (**b**) Freundlich model (m = 5 mg, V = 10 mL).

With the initial TCH concentration increased, the TCH equilibrium adsorption capacity of Eu(BTC) gradually increased, and then tended to balance (Fig. 10). Meanwhile, it was not hard to find that the temperature has a remarkable influence on TCH adsorption capacity, and high temperature benefits the TCH uptake. The q_e_–C_e_ curve ascended as the temperature increased, confirming that the TCH adsorption of Eu(BTC) was a distinct endothermic process. The theoretical maximum adsorption amount calculated by the Langmuir isotherm model was up to 397.65 mg/g at 303 K, which was higher than those of many adsorbents reported (Table S1). Compared with the results computed by Langmuir model, the Freundlich model provided a larger R^2^ value, suggesting that the multi-layer adsorption existed in the TCH adsorption of Eu(BTC). The results of Dubinin-Radushkevich model were presented in Fig. S2, and the corresponding parameters obtained were listed in Table S2. When the E value is between 8 and 16 kJ/mol, the sorption mechanism is assigned to the ion exchange process, and in the case of lower E value (less than 8 kJ/mol), it is the physical sorption^50^. The E values calculated in this study were 0.1066 to 0.2259, suggesting that the TCH adsorption processes of Eu(BTC) was controlled by physical adsorption.

### Effect of temperature: adsorption thermodynamics

In order to examine the temperature effect on TCH adsorption of Eu(BTC), the isothermal adsorption experiments were carried out at different temperatures (298, 313, 323, 333 and 343 K), and the TCH adsorption thermodynamics of Eu(BTC) were also analyzed. The main thermodynamic parameters were as follows: standard enthalpy (ΔH°), Gibbs free energy change (ΔG°) and standard entropy (ΔS°). The thermodynamic parameters were calculated according to the following Eqs. (7) and (8):

7 $$\Delta G^{ \circ } = - RT\ln \left( {K_{d} } \right)$$

8 $$\ln K_{d} = \frac{{\Delta S^{ \circ } }}{R} - \frac{{\Delta H^{ \circ } }}{RT}$$

where K_d_ means the distribution coefficient, ΔS° (J/mol·K) represents the entropy variation, R (8.314 J/mol/K) means the universal constant of ideal gases, ΔH° (kJ/mol) is the enthalpy change, T (K) represents the absolute temperature, and ΔG° (kJ/mol) is the Gibbs free energy variation^51^, respectively.

The thermodynamic parameters obtained were summarized in Table S3. As displayed in Fig. 8, the TCH adsorption capacity of Eu(BTC) continuously increased when the temperature rose. The ΔG of the system was less than zero (− 6.0229 to − 0.4070 kJ/mol), indicating that the TCH adsorption process was spontaneous. The ΔH was bigger than zero (0.1413 kJ/mol), suggesting that the TCH adsorption of Eu(BTC) was endothermic, that is, increasing the temperature could improve the adsorption performance^19^. The ΔS was 0.001840 kJ/mol/K, demonstrating the excellent binding affinity between the Eu(BTC) and TCH, and the increased disorder at the solid–liquid interface during the TCH adsorption process.

### Reusability of Eu(BTC)

The reusability is a significant factor in evaluating the practical application of adsorbents. The recyclability of Eu(BTC) was examined, as can be seen in Fig. S3. After saturated with TCH, the adsorbent was desorbed and then re-employed for TCH capture. The TCH adsorption was conducted for four times, and then no obvious decline of uptake (less than 2.87%) was observed, indicating the excellent reusability of Eu(BTC). As displayed in Fig. S4, the positions of Eu(BTC) diffraction peaks remained constant after four adsorption and desorption cycles, confirming the outstanding stability of Eu(BTC). It makes the Eu(BTC) obtained highly promising in practical application such as the TCH removal from aqueous solutions for environmental remediation.

## Conclusions

In this work, we reported an efficient and environmental-friendly method for the synthesis of Eu(BTC). The pseudo-second-order adsorption model was used to investigate the interaction between TCH and Eu(BTC), and the existence of chemical adsorption in the process of TCH capture was confirmed. The fitting curves of the Freundlich isotherm model exhibited better linearity than that of Langmuir isotherm model, indicating that the TCH adsorption of Eu(BTC) was a heterogeneous process. The maximum TCH uptake of Eu(BTC) was up to 397.65 mg/g, which was much higher than most benchmark adsorbents reported. The negative value of ΔG and the positive value of ΔH revealed the spontaneous and endothermic nature of the TCH adsorption process on Eu(BTC). The experimental facts demonstrated that the TCH adsorption mechanism of Eu(BTC) was the coexistence of π–π* interaction and chemisorption. The excellent TCH adsorption capacity and reusability of Eu(BTC) sheds a new light on antibiotics removal from wastewater.

## Supplementary Information

Supplementary Information.

## Data Availability

All data generated or analyzed during this study are included in this published article and its supplementary information file.
