# Supplementary material for: Facile fabrication of Eu-based metal–organic frameworks for highly efficient capture of tetracycline hydrochloride from aqueous solutions
Source: Sci Rep. 2023 Jul 10;13:11107. doi: 10.1038/s41598-023-38425-x (PMC10333388; doi:10.1038/s41598-023-38425-x)
Supplement: Supplementary file 1 — Supplementary Information. [file 41598_2023_38425_MOESM1_ESM.doc]

**Supplementary Material**

Facile fabrication of Eu-based metal-organic frameworks for highly efficient capture of tetracycline hydrochloride from aqueous solutions

Xue He^1,2^, Yong Liu^1,2^, Qicui Wang^1^, Tao Wang^1^, Jieli He^1^, Anzhong Peng^1, *^, Kezhen Qi^1, *^

^1^College of Pharmacy, Dali University, Dali 671003, P. R. China. ^2^These authors contributed equally: Xue He, Yong Liu. ^*^email: [penganzhong@dali.edu.cn](mailto:penganzhong@dali.edu.cn); qkzh2003@aliyun.com

**
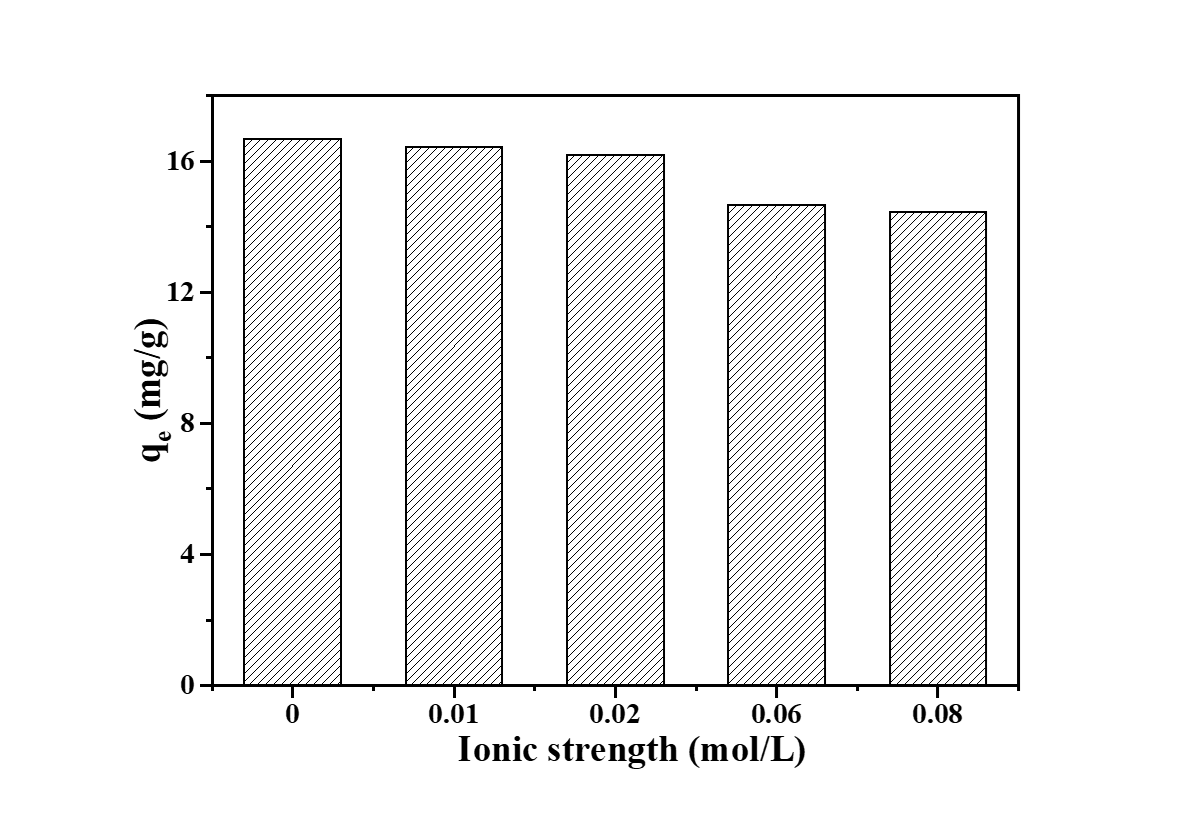
**

Fig. S1 Effect of Ionic Strength (C_0_=50 mg/L, m=20 mg, V=20 ml, T=298 K).

**
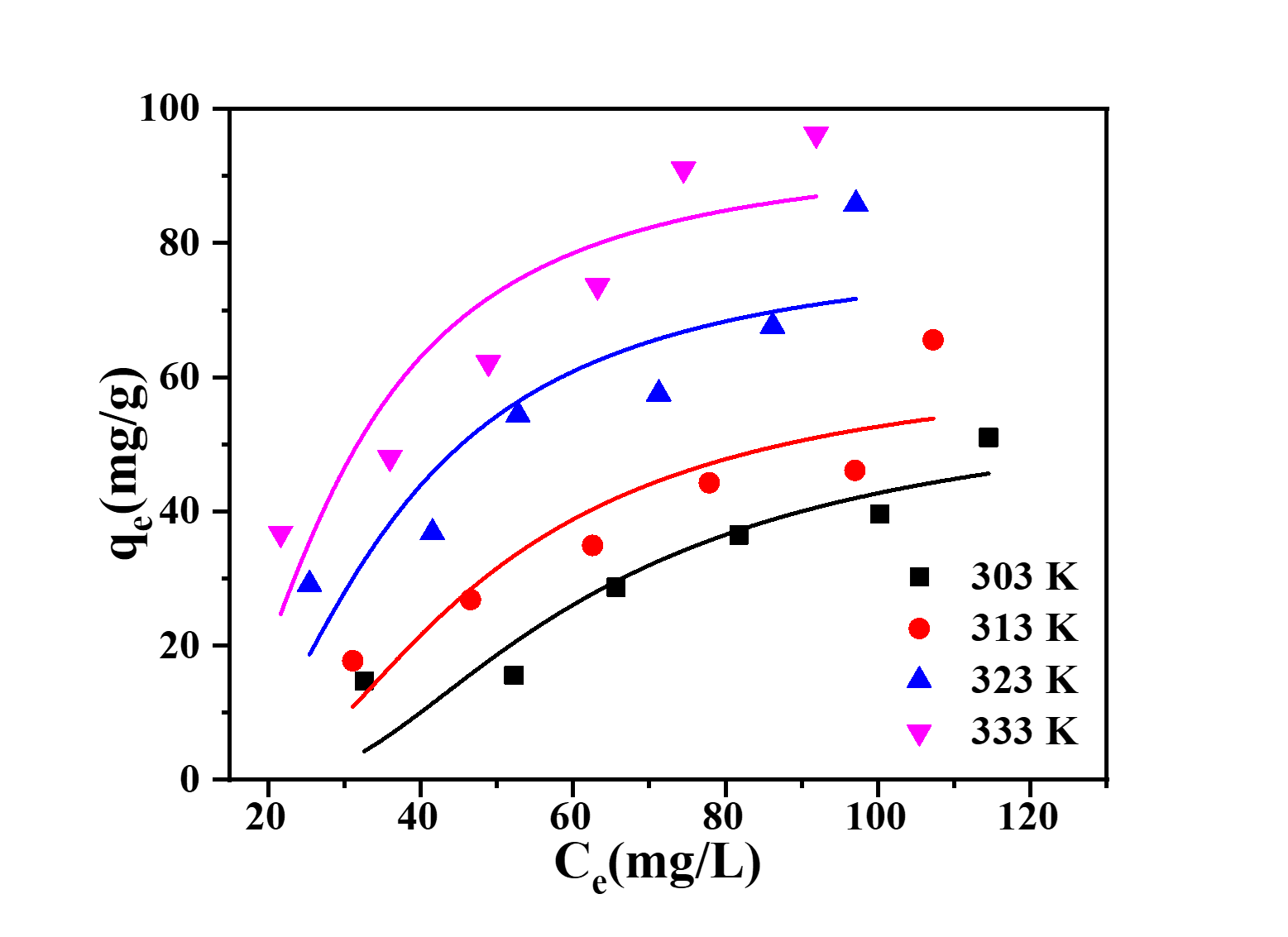
**

Fig. S2 Isotherm model fitting curves of Dubinin-Radushkevich (m=5 mg, V=10 ml).

**
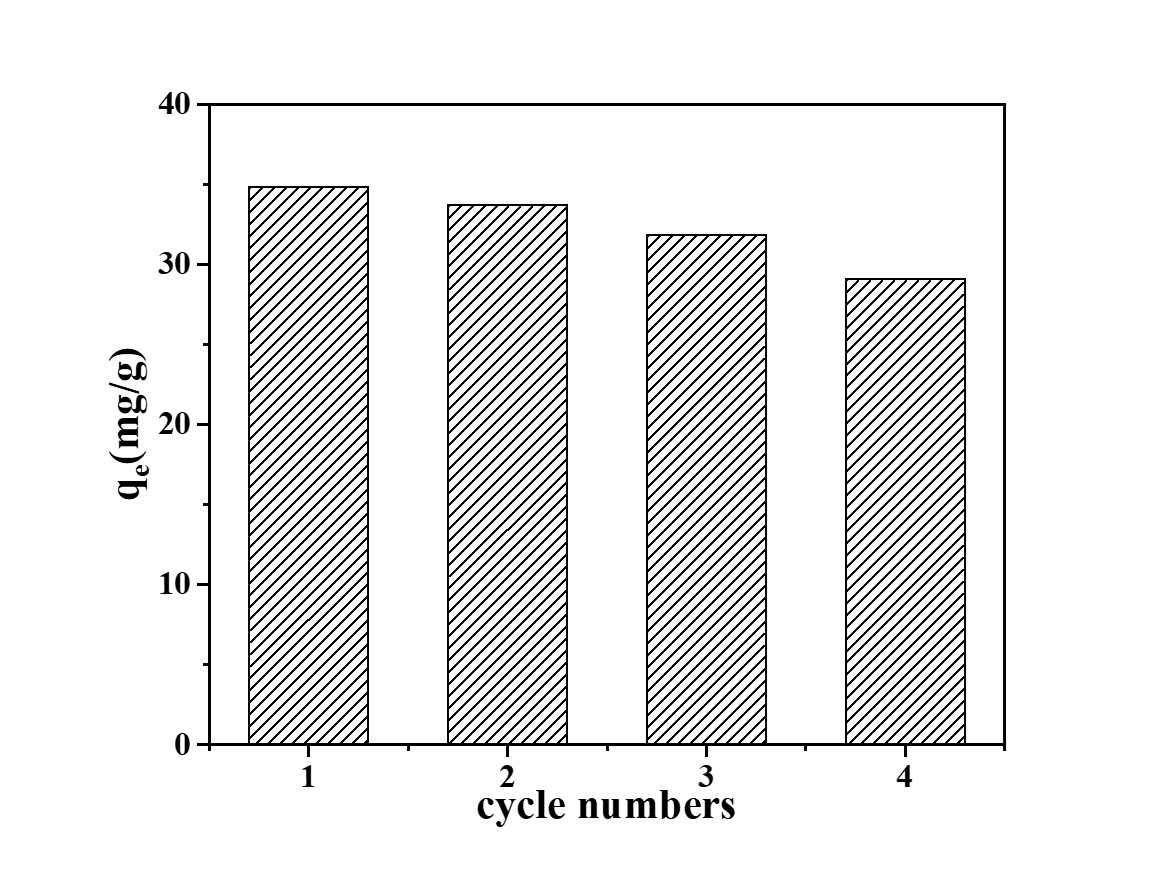
**

Fig. S3. Reusability of Eu(BTC) on TCH adsorption (C_0_=100 mg/L, m=50 mg, V=100 ml, T=298 K).

**
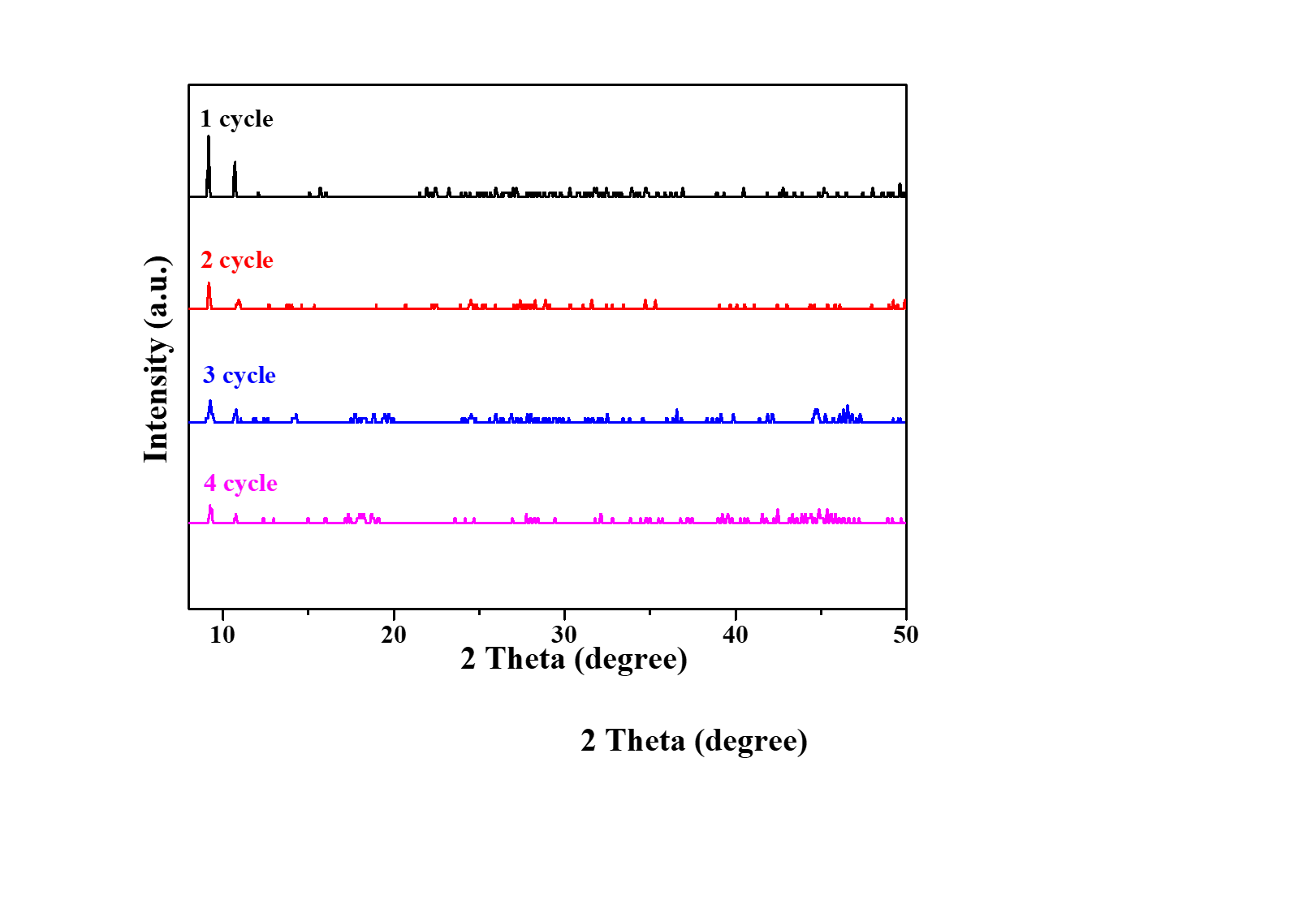
**

Fig. S4 The XRD patterns of as-synthesized Eu(BTC) for four adsorption-desorption cycles.

Table S1 The TCH adsorption capacity of different materials.

| **Materials** | ***S_BET_* (** **m^2^/g)** | **TCH uptake (** **mg/g)** | **References** |
| --- | --- | --- | --- |
| Mag-SBE@C | 122.05 | 114.45 | [1] |
| UiO-66/PDA/BC | 454 | 184.30 | [2] |
| ACS-RGO | 493.5 | 132.90 | [3] |
| x-mAC | 809.94 | 179.80 | [4] |
| MPSNMs | 2.16-2.77 | 53.29 | [5] |
| IExDEAn | / | 50.25 | [6] |
| PDA-NFsM | 5 | 161.30 | [7] |
| MSABC | 140.08 | 98.33 | [8] |
| α-Fe/Fe_3_C composite | 194.11 | 161.18 | [9] |
| BM-biochar | 257.50 | 84.54 | [10] |
| Eu(BTC) | 123.87 | 397.65 | This work |

Table S2 The parameters for TCH adsorption of Eu(BTC) by the Dubinin–Radushkevich model.

| **model** | **parameters** | **303K** | **313K** | **323K** | **333K** |
| --- | --- | --- | --- | --- | --- |
| **Dubinin–Radushkevich** | β(mol^2^/kJ^2^) | 0.000044 | 0.000033 | 0.0000042 | 0.0000098 |
|  | q_m_(mg/g) | 65.19 | 70.66 | 88.43 | 102.53 |
|  | R^2^ | 0.7848 | 0.7676 | 0.7253 | 0.7715 |
|  | E (kJ/mol) | 0.1066 | 0.1231 | 0.3450 | 0.2259 |

Table S3 thermodynamic parameters for adsorption of TCH onto Eu(BTC) magnetic microspheres.

| Antibiotic | ΔG(kJ/mol) | | | | | ΔH  (kJ/mol) | ΔS  (KJ/molK) |
| --- | --- | --- | --- | --- | --- | --- | --- |
|  | 298K | 313K | 323K | 333K | 343K |  |  |
| TCH | -0.4070 | -0.4346 | -3.3112 | -4.3181 | -6.0229 | 0.1413 | 0.00184 |

**References**

[1] Liu Y, Li J, Wu L, et al. Magnetic spent bleaching earth carbon (Mag-SBE@C) for efficient adsorption of tetracycline hydrochloride: Response surface methodology for optimization and mechanism of action [J]. Sci Total Environ, 2020, 722: 137817.

[2] Cui J, Xu X, Yang L, et al. Soft foam-like UiO-66/Polydopamine/Bacterial cellulose composite for the removal of aspirin and tetracycline hydrochloride [J]. Chemical Engineering Journal, 2020, 395.

[3] Haghighat G A, Saghi M H, Anastopoulos I, et al. Aminated graphitic carbon derived from corn stover biomass as adsorbent against antibiotic tetracycline: Optimizing the physicochemical parameters [J]. Journal of Molecular Liquids, 2020, 313.

[4] Yang Z, Zhao Z, Yang X, et al. Xanthate modified magnetic activated carbon for efficient removal of cationic dyes and tetracycline hydrochloride from aqueous solutions [J]. Colloids and Surfaces A: Physicochemical and Engineering Aspects, 2021, 615.

[5] Shan H, Si Y, Yu J, et al. Facile access to highly flexible and mesoporous structured silica fibrous membranes for tetracyclines removal [J]. Chemical Engineering Journal, 2021, 417.

[6] Zaharia M-M, Vasiliu A-L, Trofin M-A, et al. Design of multifunctional composite materials based on acrylic ion exchangers and CaCO3 as sorbents for small organic molecules [J]. Reactive and Functional Polymers, 2021, 166.

[7] Jian N, Dai Y, Wang Y, et al. Preparation of polydopamine nanofibers mat as a recyclable and efficient adsorbent for simultaneous adsorption of multiple tetracyclines in water [J]. Journal of Cleaner Production, 2021, 320.

[8] Dai J, Meng X, Zhang Y, et al. Effects of modification and magnetization of rice straw derived biochar on adsorption of tetracycline from water [J]. Bioresour Technol, 2020, 311: 123455.

[9] Jung K-W, Kim J-H, Choi J-W. Synthesis of magnetic porous carbon composite derived from metal-organic framework using recovered terephthalic acid from polyethylene terephthalate (PET) waste bottles as organic ligand and its potential as adsorbent for antibiotic tetracycline hydrochloride [J]. Composites Part B: Engineering, 2020, 187.

[10] Xiang W, Wan Y, Zhang X, et al. Adsorption of tetracycline hydrochloride onto ball-milled biochar: Governing factors and mechanisms [J]. Chemosphere, 2020, 255: 127057.
